# Cord blood fatty acid binding protein 4 and lipids in infants born small- or large-for-gestational-age

**DOI:** 10.3389/fped.2023.1078048

**Published:** 2023-05-19

**Authors:** Xin Liu, Tao Zheng, Min-Yi Tao, Rong Huang, Guang-Hui Zhang, Meng-Nan Yang, Ya-Jie Xu, Wen-Juan Wang, Hua He, Fang Fang, Yu Dong, Jian-Gao Fan, Jun Zhang, Fengxiu Ouyang, Fei Li, Zhong-Cheng Luo

**Affiliations:** ^1^ Ministry of Education-Shanghai Key Laboratory of Children’s Environmental Health, Early Life Health Institute, and Department of Pediatrics, Xinhua Hospital, Shanghai Jiao-Tong University School of Medicine, Shanghai, China; ^2^ Lunenfeld-Tanenbaum Research Institute, Prosserman Centre for Population Health Research, Department of Obstetrics and Gynecology, Mount Sinai Hospital, Temerty Faculty of Medicine, University of Toronto, Toronto, Canada; ^3^ Department of Obstetrics and Gynecology, Xinhua Hospital, Shanghai Jiao-Tong University School of Medicine, Shanghai, China; ^4^ Department of Clinical Assay Laboratory, Xinhua Hospital, Shanghai Jiao-Tong University School of Medicine, Shanghai, China; ^5^ Clinical Skills Center, School of Clinical Medicine, Shandong First Medical University & Shandong Academy of Medical Sciences, Jinan, China; ^6^ Center for Fatty Liver, Shanghai Key Lab of Pediatric Gastroenterology and Nutrition, Department of Gastroenterology, Xinhua Hospital, Shanghai Jiao Tong University School of Medicine, Shanghai, China

**Keywords:** cord blood, fatty acid binding protein 4, lipids, large for gestational age, small for gestational age, HDL, LDL

## Abstract

**Aim:**

Adverse (poor or excessive) fetal growth “programs” an elevated risk of type 2 diabetes. Fatty acid binding protein 4 (FABP4) has been implicated in regulating insulin sensitivity and lipid metabolism relevant to fetal growth. We sought to determine whether FABP4 is associated with poor or excessive fetal growth and fetal lipids.

**Methods:**

In a nested case-control study in the Shanghai Birth Cohort including 60 trios of small-for-gestational-age (SGA, an indicator of poor fetal growth), large-for-gestational-age (LGA, an indicator of excessive fetal growth) and optimal-for-gestational-age (OGA, control) infants, we measured cord blood concentrations of FABP4 and lipids [high-density lipoprotein (HDL) and low-density lipoprotein (LDL) cholesterols, triglycerides (TG)].

**Results:**

Adjusting for maternal and neonatal characteristics, higher cord blood FABP4 concentrations were associated with a lower odds of SGA [OR = 0.29 (0.11–0.77) per log unit increment in FABP4, *P* = 0.01], but were not associated with LGA (*P* = 0.46). Cord blood FABP4 was positively correlated with both LDL (*r* = 0.29, *P* = 0.025) and HDL (*r* = 0.33, *P* = 0.01) in LGA infants only.

**Conclusion:**

FABP4 was inversely associated with the risk of SGA. The study is the first to demonstrate LGA-specific positive correlations of cord blood FABP4 with HDL and LDL cholesterols, suggesting a role of FABP4 in fetal lipid metabolism in subjects with excessive fetal growth.

## Introduction

Fatty acid binding protein 4 (FABP4) is an adipokine that binds to saturated and unsaturated fatty acids (1). FABP4 is mainly expressed in adipocytes, but also in macrophages at lower amounts (1). FABP4 expression is highly induced during adipocyte differentiation and affected by fatty acids, insulin and peroxisome proliferator activated receptor γ agonists (2). In obese FABP4-deficient mice, the conversion of glucose to fatty acids is elevated two-folds, suggesting FABP4 may suppress lipogenesis (3). Treatment with recombinant FABP4 induces insulin resistance in mice (4), suggesting a role of FABP4 in regulating insulin sensitivity. A recent study suggests that FABP4 may be involved in the regulation of neonatal glucose homeostasis in humans (5). Elevated circulating FABP4 concentrations have been associated with insulin resistance in adults (6). It is unclear whether FABP4 affects fetal growth which is influenced by insulin sensitivity.

FABP4 regulates cellular lipid metabolism and the reception of lipid signals relevant to the atherogenic processes (7). FABP4-deficiency may protect against atherosclerosis in apolipoprotein E-deficient mice, and FABP4 knockout mice may have a reduced ability in accumulating cholesterol esters (8). FABP4 in macrophages may increase the accumulation of cholesterol ester and foam cell formation (9). A study in mouse pre-adipocytes suggests that exogenous FABP4 may interfere with adipocyte differentiation and induce P38/HSL mediated lipolysis (10). FABP4 may regulate intracellular lipid accumulation in human trophoblasts (11). It is unknown whether FABP may affect lipid/cholesterol metabolism in human fetuses.

Poor or excessive fetal growth “programs” an elevated risk of obesity and type 2 diabetes in later life (12). It remains unclear whether FABP4 is involved in poor or excessive fetal growth. We are aware of only three studies. A Danish birth cohort study has associated low birth weight with low FABP4 mRNA expression in adipocytes (13), suggesting an association between FABP4 and fetal growth. However, both higher and lower cord blood FABP4 levels have been reported in small-for-gestational-age (SGA, an indicator of poor fetal growth) relative to normal birth weight infants (14, 15).

The goals of this study were to determine whether FABP4 is associated with poor or excessive fetal growth, and whether FABP4 is correlated with fetal lipids (cholesterols) in normal, poor or excessive fetal growth.

## Methods

### Study population

This was a nested matched case-control study in the Shanghai Birth Cohort (SBC) (16). The SBC is a prospective cohort recruiting expectant women in Shanghai 2013–2016. The women were followed up at the second and third trimesters of pregnancy and delivery. Cord blood specimens were collected at delivery. The study was approved by the research ethics committees of Xinhua Hospital (the coordination center, ref # M2013-010) and all participating hospitals. Written informed consent was obtained from all study participants.

There were 3,692 singleton live births in the SBC. Infants were classified as SGA (birth weight <10th percentile), appropriate-for-gestational-age (AGA, 10th–90th percentiles) and large-for-gestational-age (LGA, >90th percentile) according to the 2015 Chinese sex- and gestational age-specific birth weight standards (17). Among the AGA infants, we defined optimal-for-gestational-age (OGA) as birth weight between 25th and 75th percentiles, for the purpose of contrasting infants with poor (SGA) or excessive (LGA) fetal growth to optimal fetal growth.

The nested case-control fetal growth study is a random sample of 60 trios of SGA, LGA and OGA (control) singleton infants matched by sex (the same) and gestational age at birth (within 1 week) in the SBC as has been described recently (18). Eligible subjects must meet all the following criteria: (1) Han ethnicity (the majority ethnic group, >98%); (2) maternal age 20–45 years; (3) natural conception; (4) singleton pregnancy; (5) no maternal severe chronic disease or severe pregnancy complications (e.g., diabetes, heart disease, preeclampsia or eclampsia); (6) no birth defects; (7) 5 min Apgar score ≥7; (8) cord blood specimen available for biomarker assays. All mothers were non-smokers.

### Specimens and assays

Cord blood serum (without anticoagulant) and EDTA plasma samples were obtained by centrifugation at 4°C, 4,000 rpm for 10 min, and stored in multiple aliquots at −80°C until assays. The assay technicians were blinded to the clinical status (SGA, LGA or OGA) of study subjects.

Cord plasma FABP4 was measured by an enzyme-linked immunosorbent assay (ELISA) kit (R&D systems, Minnesota, USA). Cord serum levels of TG, LDL and HDL and total cholesterol (TC) were determined using a Hitachi 7600 Automatic Biochemistry Analyzer.

As reported previously (18), cord plasma proinsulin was measured by an ELISA kit from Mercodia (Uppsala, Sweden), and IGF-II by an ELISA kit from R&D system (Minneapolis, USA), and the absorbance was determined using a microplate spectrophotometer (Beckman CX7, USA). Cord serum insulin and IGF-I concentrations were detected by automated chemiluminescent assays (ADVIA Centaur and Immulite 2000, SIEMENS, Germany). An acid buffer dilution was used to release the IGF binding protein bound IGFs in the measurements of IGF-I and IGF-II.

The minimal detectable concentrations were 0.007 ng/ml for FABP4, 0.011 mmol/L for TG, 0.026 mmol/L for LDL, 0.026 mmol/L for HDL, 0.026 mmol/L for TC, 3.5 pmol/L for insulin, 1.7 pmol/L for proinsulin, 25 ng/ml for IGF-I and 1.88 pg/ml for IGF-II, respectively. The intra-assay and inter-assay coefficients of variation were in the ranges of 3.4%–12.7% for FABP4, 0.5%–2.0% for TG, LDL, HDL and TC, 2.0%–6.5% for insulin and IGF-I, 0.3%–5.0% for proinsulin and 2.4%–9.3% for IGF-II, respectively.

### Statistical analysis

Continuous variables are presented as mean ± SD. Biomarker data were log-transformed in correlation and regression analyses. Birth weight *z* score was calculated according to the 2015 Chinese sex- and gestational age-specific birth weight standards (17). Cord blood glucose-to-insulin ratio was calculated as an indicator of fetal insulin sensitivity (19). Pearson partial correlation coefficients were calculated in evaluating the associations of cord blood FABP4 with cholesterols and fetal growth factors adjusting for gestational age at delivery. Fisher's *z* test was used in the comparisons of correlation coefficients. Generalized linear model was used to assess the differences in cord blood biomarkers adjusting for maternal and neonatal characteristics. Multinomial logistic regression was used to assess whether cord blood FABP4 was predictive of SGA or LGA adjusting for maternal and neonatal characteristics. Only co-variables with *P* < 0.2 were included in the parsimonious final multivariate models. Statistical analyses were performed using SAS Version 9.4 (SAS Institute, Cary, NC, USA).

Using an online sample size and power calculator (http://powerandsamplesize.com/Calculators/), we estimated that with the study sample sizes (60 SGA, 60 LGA and 60 OGA) and type 1 error (alpha) at 2.5%, the study had a power of 94% to detect a 0.7 SD or greater difference in cord plasma FABP4 concentration (the primary outcome) comparing SGA or LGA to OGA infants. *P* < 0.025 was considered statistically significant in assessing the differences in cord plasma FABP4 concentration comparing SGA or LGA vs. OGA infants (Bonferroni correction for 2 tests). *P* < 0.05 was considered statistically significant in assessing the correlations between cord blood FABP4 and cholesterols, and in exploring the predictors of SGA and LGA in order to capture all potentially important predictors.

## Results

### Participants' characteristics

Maternal and neonatal characteristics in this nested case-control fetal growth study in the SBC have been described recently (Supplementary Appendix Table S1) (18). Briefly, there were no significant differences in maternal age, education, parity, family history of diabetes and gestational age at delivery. LGA newborns had higher maternal pre-pregnancy BMI (mean: 22.4 vs. 21.2 kg/m^2^), and were more frequently delivered by cesarean section (58% vs. 20%) than OGA newborns. SGA infants had lower maternal pre-pregnancy BMI (mean: 20.1 vs. 21.2 kg/m^2^), but did not differ from OGA infants in other maternal characteristics. The mothers of OGA infants more frequently drank alcohol during pregnancy than the mothers of SGA infants (13.0% vs. 1.7%). Maternal fasting glucose concentrations at 24–28 weeks of gestation were higher in LGA vs. OGA groups (mean: 4.6 vs. 4.4 mmol/L) (18).

### Cord blood biomarkers

Comparing cesarean section vs. vaginal deliveries, cord blood FABP4 concentrations were higher (mean ± SD: 25.1 ± 16.4 vs.17.4 ± 8.9 ng/ml, *P* = 0.0002), and TG concentrations lower (0.24 ± 0.21 vs. 0.29 ± 0.14 mmol/L, *P* = 0.002), while there were no differences in cord blood LDL, HDL and total cholesterol concentrations.

Adjusting for pre-pregnancy BMI, primiparity and cesarean section (other factors did not affect the comparisons), cord blood FABP4 concentrations were progressively higher from SGA to OGA to LGA infants (regression test for trends, *P* = 0.02), and were significantly lower in SGA vs. OGA infants (*P* = 0.02), but did not differ significantly between LGA and OGA infants (Table 1). Cord blood LDL concentrations were lower in LGA relative to OGA infants, but did not differ between SGA and OGA infants. Cord blood TG concentrations were progressively lower from SGA to OGA to LGA infants (regression test for trends, *P* < 0.01). Cord blood HDL concentrations were lower in SGA vs. OGA infants (*P* = 0.02), but did not differ between LGA and OGA infants. There were no significant differences in cord blood total cholesterol concentrations between SGA or LGA vs. OGA infants. Cord blood glucose-to-insulin ratios [higher values indicating better insulin sensitivity (19)] were progressively lower from SGA to OGA to LGA infants (*P* < 0.01).

**Table 1:** Cord blood FABP4, lipids, glucose, insulin and glucose-to-insulin ratio in SGA, OGA and LGA infants.

	SGA (*n* = 60)	OGA (*n* = 60)	LGA (*n* = 60)	*P* ^1^	*P* ^2^	*P* ^3^
FABP4, ng/ml	18.8 ± 14.5	20.4 ± 11.2	22.2 ± 13.5	**0** **.** **02**	0.40	**0**.**02**
LDL, mmol/L	0.52 ± 0.35	0.55 ± 0.29	0.43 ± 0.21	0.73	**0**.**01**	0.06
HDL, mmol/L	0.85 ± 0.32	0.96 ± 0.32	0.93 ± 0.25	**0**.**02**	0.95	**0**.**045**
TG, mmol/L	0.33 ± 0.17	0.26 ± 0.12	0.22 ± 0.20	**<0**.**01**	**0**.**045**	**<0**.**01**
TC, mmol/L	1.73 ± 0.46	1.85 ± 0.56	1.69 ± 0.46	0.40	0.35	0.62
Glucose-to-insulin ratio, mg/dl/mU/L	32.0 ± 27.7	25.3 ± 19.3	17.5 ± 19.2	**0**.**03**	0.11	**<0**.**01**

Data presented are mean centerSD.

Unit conversion: TC, HDL and LDL: 1 mmol/L = 38.67 mg/dl; TG 1 mmol/L = 88.57 mg/dl; glucose 1 mmol/L = 18 mg/dl; insulin 1 mU/L = 6 pmol/L.

FABP4, fatty acid binding protein 4; SGA, small-for-gestational-age (birth weight <10th percentile); OGA, optimal-for-gestational-age (25th-75th percentiles); LGA, large-for-gestational-age (>90th percentile); TG, triglyceride; LDL, low density lipoprotein; HDL, high density lipoprotein; TC, total cholesterol.

*P*^1^ values comparing SGA vs. OGA; *P*^2^ values comparing LGA vs. OGA adjusting for pre-pregnancy BMI, primiparity and cesarean section (other maternal and neonatal characteristic co-variables were excluded at *P* > 0.2; they did not affect the comparisons) in generalized linear models; *P*^3^ values in regression tests for trends in each biomarker across the 3 groups (SGA to OGA to LGA); log-transformed biomarker data were used in regression models.

*P* values in bold, *P* < 0.05.

As reported previously, cord blood insulin concentrations were progressively lower, while proinsulin, IGF-I and IGF-II concentrations progressively higher from SGA to OGA to LGA infants (18).

### Correlations

Adjusting for gestational age at delivery, cord blood FABP4 was positively correlated with proinsulin, birth weight (*z* score), and negatively correlated with gestational age in the total study sample (Table 2). Cord blood FABP4 and birth weight (*z* score) were positively correlated in LGA infants (*r* = 0.28, *P* = 0.03), but towards negatively correlated in SGA infants (*r* = −0.23, *P* = 0.10) (Fisher's *z* test for difference in r, *P* = 0.01). Cord blood FABP4 was positively correlated with LDL (*r* = 0.29, *P* = 0.03), HDL (*r* = 0.33, *P* = 0.01) and TC (*r* = 0.38, *P* = 0.003) in LGA infants only.

**Table 2:** Correlations of cord blood FABP4 with gestational age, birth weight (*z* score), fetal growth factors, TG, LDL, HDL, TC and glucose-to-insulin ratio (an indicator of fetal insulin sensitivity) in all, SGA, OGA and LGA infants.

	ALL	SGA	OGA	LGA	SGA vs. OGA	LGA vs. OGA	LGA vs. SGA
FABP4 with:	*r*	*r* ^1^	*r* ^2^	*r* ^3^	*P* ^1^	*P* ^2^	*P* ^3^
Gestational age	**−0** **.** **16** *	−0.09	−0.14	**−0**.29*	0.79	0.42	0.29
Birth weight *z*	**0**.16*	−0.23	−0.05	**0**.28*	0.34	0.08	**0**.**01**
Insulin	0.06	−0.12	0.08	0.12	0.30	0.84	0.22
Proinsulin	**0**.21**	0.10	0.17	0.20	0.72	0.86	0.60
IGF-I	0.11	0.01	−0.14	0.17	0.47	0.10	0.43
IGF-II	−0.07	−0.10	−0.14	−0.13	0.84	0.95	0.89
TG	−0.08	−0.21	0.02	0.21	0.22	0.31	**0**.**03**
LDL	0.00	−0.19	0.01	**0**.29*	0.31	0.12	**0**.**01**
HDL	−0.04	−0.24	−0.02	**0**.33*	0.25	0.053	**<0**.**01**
TC	0.03	−0.25	0.03	**0**.38**	0.14	**0**.**048**	**<0**.**01**
Glucose-to-insulin ratio	−0.08	0.02	−0.02	−0.05	0.86	0.88	0.76

Data presented are Pearson partial correlations adjusting for gestational age at delivery/blood sampling; log-transformed data were used for biomarkers in the correlation analyses.

*r*^1^, *r*^2^, *r*^3^ were correlation coefficients in SGA, OGA and LGA infants, respectively.

FABP4, fatty acid binding protein 4; SGA, small-for-gestational-age (birth weight <10th percentile); OGA, optimal-for-gestational-age (25th–75th percentiles); LGA, large-for-gestational-age (>90th percentile); IGF, insulin-like growth factor;.

TG, triglyceride; LDL, low density lipoprotein; HDL, high density lipoprotein; TC, total cholesterol.

*P*^1^ values comparing SGA and OGA groups, *P*^2^ values comparing LGA and OGA groups, *P*^3^ values comparing LGA and SGA groups, in Fisher's *z* tests for differences in correlation coefficients.

* *P* < 0.05;

** *P* < 0.01; *P* values in bold, *P* < 0.05.

### Associations of cord blood FABP4 with SGA and LGA

Adjusting for pre-pregnancy BMI, primiparity and cesarean section, higher cord blood FABP4 concentrations were associated with a lower odds of SGA (OR = 0.31) (Table 3). Cord blood FABP4 was not associated with LGA. Higher cord blood LDL levels were associated with a lower odds of LGA (OR = 0.37). Higher cord blood TG levels were associated with a higher odds of SGA (OR = 3.82), while higher cord blood HDL levels were associated with a lower odds of SGA (OR = 0.23).

**Table 3:** Associations of cord blood FABP4 and cholesterols with SGA and LGA.

	Crude OR		Adjusted OR*	
	(95% CI)	*P*	(95% CI)	*P*
**FABP4**				
SGA	0.52 (0.25, 1.08)	0.08	0.31 (0.12, 0.77)	**0** **.** **01**
LGA	1.24 (0.64, 2.42)	0.52	0.69 (0.30, 1.55)	0.37
**LDL**				
SGA	0.78 (0.38, 1.61)	0.50	0.84 (0.38, 1.85)	0.66
LGA	0.39 (0.18, 0.81)	**0**.**01**	0.37 (0.16, 0.84)	**0**.**02**
**HDL**				
SGA	0.27 (0.08, 0.92)	**0**.**04**	0.23 (0.06, 0.93)	**0**.**04**
LGA	0.80 (0.25, 2.63)	0.72	1.37 (0.33, 5.60)	0.66
**TG**				
SGA	2.72 (1.25, 5.92)	**0**.**01**	3.82 (1.48, 9.89)	**<0**.**01**
LGA	0.34 (0.15, 0.79)	**0**.**01**	0.38 (0.14, 1.01)	0.052
**TC**				
SGA	0.48 (0.13, 1.79)	0.27	0.50 (0.11, 2.21)	0.36
LGA	0.34 (0.09, 1.29)	0.11	0.48 (0.10, 2.19)	0.34

Data presented are the odds ratios (OR) of SGA or LGA (relative to OGA) per log unit increment in FABP4 or cholesterols from multinomial logistic regression models.

*P* values in bold, *P* < 0.05.

* Adjusted for pre-pregnancy BMI, primiparity and cesarean section; other maternal and neonatal characteristic co-variables were excluded at *P* > 0.2 (they did not affect the comparisons).

FABP4, fatty acid binding protein 4; SGA, small-for-gestational-age (<10th percentile); LGA, large-for-gestational-age (>90th percentile), OGA, optimal-for-gestational-age (25th-75th percentiles); TG, triglyceride; LDL, low density lipoprotein; HDL, high density lipoprotein; TC, total cholesterol.

In the fully adjusted model including pre-pregnancy BMI, primiparity, cesarean section and cord blood TG, HDL and LDL (Table 4), the ORs for cord blood FABP4 in association with SGA or LGA were in general similar compared to the ORs adjusting for pre-pregnancy BMI, primiparity and cesarean section only (Table 3). Higher cord blood FABP4 levels were associated with a lower odds of SGA (OR = 0.29). Higher cord blood LDL levels were associated with a lower odds of LGA (OR = 0.29), while higher TG levels were associated with a higher odds of SGA (OR = 2.94) (Table 4). Higher prepregnancy BMI was associated with a lower odds of SGA (OR = 0.83), and a higher odds of LGA (OR = 1.23). As expected, cesarean section was associated with a higher odds of LGA (OR = 3.13).

**Table 4:** Associations of maternal pre-pregnancy BMI, primiparity, cesarean section, cord blood FABP4, LDL, HDL and TG with SGA and LGA in the fully adjusted model.

	SGA		LGA	
	OR (95% CI)	*P*	OR (95% CI)	*P*
Pre-pregnancy BMI	0.83 (0.69, 0.99)	**0.04**	1.23 (1.04, 1.46)	**0.02**
Primiparity	2.61 (0.69, 9.95)	0.16	1.46 (0.50, 4.31)	0.49
Cesarean section	2.55 (0.94, 6.88)	0.06	3.13 (1.24. 7.95)	**0.02**
**Cord blood**				
FABP4	0.29 (0.11, 0.77)	**0.01**	0.73 (0.32, 1.68)	0.46
LDL	0.86 (0.31, 2.37)	0.77	0.29 (0.10, 0.82)	**0.02**
HDL	0.32 (0.08, 1.28)	0.11	3.35 (0.59, 19.02)	0.17
TG	2.94 (1.01, 8.56)	**0.047**	0.45 (0.16, 1.29)	0.14

Data presented are the adjusted odds ratios (OR) of SGA or LGA (relative to OGA) in the final parsimonious multinomial logistic model including pre-pregnancy BMI, primiparity, cesarean section, cord blood FABP4, TG, LDL and HDL; other maternal and neonatal characteristic co-variables were excluded at *P* > 0.2 (they did not affect the comparisons). The effect estimates are for per log unit increment in each biomarker.

FABP4, fatty acid binding protein 4; SGA, small-for-gestational-age (<10th percentile); LGA, large-for-gestational-age (>90th percentile);.

TG, triglyceride; LDL, low density lipoprotein; HDL, high density lipoprotein; TC, total cholesterol.

*P* values in bold, *P* < 0.05.

## Discussion

We observed that higher cord blood FABP4 concentrations were associated with a lower odds of SGA, while higher cord blood TG concentrations were associated with a higher odds of SGA. Cord blood FABP4 was positively correlated to HDL and LDL cholesterols in LGA infants, but not in SGA or OGA infants. This novel observation suggests an impact of FABP4 on fetal cholesterol/lipid metabolism exclusively in subjects with excessive fetal growth.

Data are scarce on cord blood FABP4 levels in SGA and LGA infants. We are aware of only two studies (14, 15). Consistent with our data on Chinese infants in Shanghai, Joung and colleagues reported a trend towards higher cord blood FABP4 levels from SGA (*n* = 40) to appropriate-for-gestational-age (AGA, *n* = 288) to LGA (*n* = 33) infants (*P* = 0.07, about 2/3 White) in Boston (15). In contrast, Papathanasiou and colleagues reported higher FABP4 levels at both extremes (SGA or LGA, *n* = 20 each) relative to AGA (*n* = 40) infants in Athens, Greece (14). The discrepant findings may be partly due to the differences in study population and sample size (14, 15). Each log unit increment in cord blood FABP4 was associated with about a 70% decrease in the odds of SGA in our study. Similar to a previous study (15), we did not detect an association between FABP4 and LGA.

Higher circulating FABP4 concentrations have been reported in obese and overweight relative to normal-weight children (20, 21). Similarly, there was a trend towards higher cord blood FABP4 concentrations from SGA to OGA to LGA infants in our study. SGA infants have lower FABP4 that may be attributable to lower fat mass, while LGA infants tend to have higher FABP that may be attributable to higher fat mass (22).

Total cholesterol and weight gain have been correlated with FABP4 levels in preterm infants (23). In high-fat diet-induced mice, silencing the FABP4 gene results in metabolic recovery and body weight reduction (24). These observations suggest that FABP4 may affect adipose tissue homeostasis or lipid/cholesterol metabolism. There is a lack of data on FABP4 in relation to lipids in cord blood in humans. Our study is the first to report LGA-specific positive correlations of cord blood FABP4 with LDL and HDL, suggesting that FABP4 may affect fetal lipid metabolism exclusively in subjects with excessive fetal growth. Serum FABP4 levels are predictive of the risk of metabolic syndrome (25). The positive correlations of FABP4 with LDL and HDL in LGA subjects are similar to previous observations that higher circulating FABP4 levels were correlated to higher percent body fat, LDL and HDL and total cholesterol concentrations in adults (25). FABP4 may regulate cellular lipid metabolism and accelerate the accumulation of cholesterol esters (7, 8), and promote lipolysis resulting in ectopic lipid accumulation (10), and thus may contribute to excessive fetal growth and explain the positive correlation with birth weight in LGA infants.

Both increased and decreased nourishment *in utero* may affect neonatal lipoprotein profiles (26, 27). Lipoprotein lipase hydrolyzes triacylglycerol to non-essential fatty acids which may be taken up and stored in adipose tissue (28). The activity of placental lipoprotein lipase has been positively correlated with neonatal adiposity (29). Consistent with our data, higher cord blood triglyceride levels have been reported in SGA infants (26, 30). This may be partly attributable to decreased LPL activity and impaired ability to hydrolyze triglycerides leading to reduced peripheral adipose deposition (30).

Consistent with our data, higher cord blood LDL levels were associated with a lower risk of LGA in a previous study (26), but two other studies did not detect any association (27, 31). More studies are warranted to clarify the association. We speculated that LGA infants may have enhanced capacity in metabolizing lipids, leading to lower LDL levels. Cord blood HDL concentrations were lower in SGA infants, consistent with the results in previous studies (27, 32).

Consistent with a previous study, we observed lower cord blood glucose-to-insulin ratios in LGA vs. OGA groups indicating lower insulin sensitivity in LGA newborns (19). FABP4 has been implicated in the regulation of insulin resistance in mice (4), and in the regulation of neonatal glucose homeostasis in humans (5). However, we did not observe any correlation between cord blood FABP4 and glucose-to-insulin ratio, suggesting that FABP4 may not contribute to lower fetal insulin sensitivity in LGA infants.

For better contrasts between infants with poor fetal growth (SGA) or excessive fetal growth (LGA) to normal fetal growth, we selected OGA infants (birth weight between 25th and 75th percentiles) rather than AGA infants (birth weight between 10th and 90th percentiles) as the reference. We defined fetal growth status using the 2015 Chinese sex- and gestational age-specific birth weight standards, and the birth weight cutoffs are in general higher than the Intergrowth birth weight standards (33), but lower than the WHO and Fenton birth weight standards (34, 35). For example, at 40 weeks of gestation, the 90th birth weight percentile cutoff in defining LGA in male newborns is 4,030 g by Chinese standards (17), vs. 3,940 g by Intergrowth standards (33), 4,067 g by WHO standards (34), and 4,185 g by Fenton standards (35).

Our study has some limitations. The study sample size was modest, and was powered to detect moderate to large differences (≥0.7 SD), but not powered to detect small differences. All participants were Chinese, and more studies in other ethnic groups are warranted to understand the generalizability of the study findings. The study is observational in nature. We could not draw firm inference regarding causality.

## Conclusion

Cord blood FABP4 was inversely associated with the odds of being born SGA, suggesting that low FABP4 may be a biomarker of poor fetal growth. FABP4 was positively correlated with HDL and LDL cholesterol exclusively in LGA subjects, suggesting a role of FABP4 in fetal lipid metabolism in subjects with excessive fetal growth.

## Data Availability

The datasets presented in this article are not readily available because access to the deidentified participant research data must be approved by the research ethics board on a case-by-case basis. Requests to access the datasets should be directed to Zhong-Cheng Luo, zc_luo@yahoo.com; Fei LI, feili@shsmu.edu.cn.
