# Supplementary material for: Cord blood fatty acid binding protein 4 and lipids in infants born small- or large-for-gestational-age
Source: Front Pediatr. 2023 May 19;11:1078048. doi: 10.3389/fped.2023.1078048 (PMC10237290; doi:10.3389/fped.2023.1078048)
Supplement: Supplementary file 1 [file Table1.pdf]

Table S1. Maternal and neonatal characteristics in SGA, OGA and LGA singleton newborns*

	OGA	SGA	LGA	P [1]	P [2]
**N**	60	60	60		
**Maternal characteristics**					
Age, years	30.0±3.5	29.4±3.3	28.9±3.5	0.324	0.080
>35	6 (10)	4 (6.7)	3 (5.0)	0.741	0.488
Education (university)	39 (65)	36 (60)	38 (63)	0.491	0.752
Drinking alcohol	8 (13)	1 (1.7)	2 (3.3)	0.037	0.180
Family history of diabetes	6 (10)	7 (12)	5 (8.3)	0.617	0.901
Pre-pregnancy BMI	21.2±2.7	20.1±2.5	22.4±2.6	0.058	0.009
BMI Group				0.005	0.001
<18.5	8 (13)	15 (23)	1 (2)		
18.5-24.0	45 (75)	31 (52)	36 (60)		
≥24.0	6 (10)	4 (6.7)	12 (20)		
Primiparity	47 (78)	55 (92)	49 (82)	0.074	0.820
75 g OGTT (mmol/L)					
Fasting	4.34±0.40	4.42±0.38	4.61±0.39	0.323	<0.001
1-hour	7.36±1.4	7.77±1.7	8.22±1.5	0.309	0.002
2-hours	6.28±1.0	6.53±1.6	6.77±1.2	0.252	0.069
HbA1C (%)	5.05±0.32	4.94±0.32	5.09±0.35	0.244	0.561
**Neonatal characteristics**					
C-section delivery	12 (20)	17 (28)	35 (58)	0.333	<0.001
Sex, male	33 (55)	33 (55)	33 (55)	1.00	1.00
Gestational age (weeks)	39.6±1.1	39.5±1.2	39.6±1.2	0.130	0.825
Birth weight (g)	3372±264	2674±293	4162±351	<0.001	<0.001
z score	0.13±0.65	-1.63±0.68	2.10±0.76	<0.001	<0.001
Birth length (cm)	49.8±1.2	48.7±1.5	51.2±1.1	<0.001	<0.001
z score	-0.15±0.98	-1.08±1.30	1.21±1.00	<0.001	<0.001

*Data presented are Mean±SD or n (%). The study subjects were 60 trios of SGA, OGA and LGA newborn infants matched by sex and gestational age (weeks) at delivery; there was no maternal smoker.

SGA, small-for-gestational-age (<10th percentile); OGA, optimal-for-gestational-age (25-75th h percentiles); LGA= large-for-gestational-age (>90th percentile); OGTT, oral glucose tolerance test (at 24-28 weeks of gestation).

[1] P values comparing SGA vs. OGA groups;

[2] P values comparing LGA vs. OGA groups in paired t-tests for continuous variables or chi-square tests for categorical variables.

Source: reference #18.
